# IL-1 and T Helper Immune Responses

**DOI:** 10.3389/fimmu.2013.00182

**Published:** 2013-07-15

**Authors:** Veronica Santarlasci, Lorenzo Cosmi, Laura Maggi, Francesco Liotta, Francesco Annunziato

**Affiliations:** ^1^Department of Experimental and Clinical Medicine, University of Florence, Florence, Italy

**Keywords:** Th17 cells, Th2 cells, Th1 non-classic, IL-1RI and T cells, IL-1 and T cells

## Abstract

CD4 T cells play a critical role in mediating adaptive immunity to a variety of pathogens as well as in tumor immunity. If not adequately regulated, CD4 T cells can be also involved in autoimmunity, asthma, and allergic responses. During TCR activation in a particular cytokine milieu, naïve CD4 T cells may differentiate into one of several lineages of T helper (Th) cells, including Th1, Th2, and Th17, as defined by their pattern of cytokine production and function. IL-1, the prototypic proinflammatory cytokine, has been shown to influence growth and differentiation of immunocompetent lymphocytes. The differential expression of IL-1RI on human CD4 T cell subsets confers distinct capacities to acquire specific effector functions. In this review, we summarize the role of IL-1 on CD4 T cells, in terms of differentiation, activation, and maintenance or survival.

## T Helper Cell Subsets

CD4+ T helper (Th) lymphocytes represent a heterogeneous population of cells that play an essential role in adaptive immunity. These cells include effector cells, which are devoted to protection against pathogens, and regulatory T cells (T_regs_), which protect against effector responses to autoantigens, and also to exogenous antigens when they become dangerous for the host. The term Th derived from the observation that these cells were critical for helping B cells to produce antibodies in the primary response (humoral immunity). CD4+ T cells were also found to be responsible for the so called cell-mediated immunity, or delayed-type hypersensitivity, which is characterized by reaction involving activation of macrophages. The distinct protective function of different effector CD4+ T lymphocytes, enables the best type of response according to the nature of the invading microorganism. Th1 cells produce high levels of IFN-γ and are responsible for both phagocyte activation and the production of opsonizing and complement-fixing antibodies, thus playing an important role in protection against intracellular pathogens. Th2 cells produce IL-4, IL-5, IL-9, and IL-13. Th2 cells, because of their ability to produce IL-4 and IL-13, can induce IgE class switching by B lymphocytes, enabling in this way mast cells and basophils sensitization and possible subsequent activation. In addition, IL-5 produced by Th2 cells has specific activity on differentiation, activation, and recruitment of eosinophils. Finally, IL-9 has an important role in the proliferation and accumulation of mast cells and the induction of mucus production by cells of the respiratory tract and the gut (1–4). Because of all the mentioned characteristics, Th2 cells are effective in the protection against helminthes (5). The more recently discovered Th17 subset is characterized by the production of IL-17A, IL-17F, IL-8, IL-21, and IL-22. Th17 cells play a critical role in the recruitment, activation, and migration of neutrophil granulocytes, both directly, through IL-8 production (6) and indirectly, by inducing, via IL-17, the production of colony stimulatory factors (CSF) and CXCL8 (7) in tissue resident cells. Because of their unique ability to recruit neutrophils, the main protective function of Th17 cells appears to be the clearance of extracellular pathogens, including fungi (8, 9). The distinctive features of the various CD4 effector/regulatory subpopulations are determined largely by the set of transcription factors they express and the genes they transcribe. The induction of the distinctive patterns of gene expression is dependent on the milieu of microenvironmental cytokines during the antigen-mediated activation of a naïve T cell.

In addition to their protective functions against invading pathogens, Th1, Th2, and Th17 cells contribute to the development of human disorders: Th1 and Th17 cells have been thought to be involved in the pathogenesis of organ-specific autoimmune diseases, as well as other chronic inflammatory disorders, such as Crohn’s disease (CD), psoriasis, and rheumatoid arthritis (RA); Th2 cells certainly play a central role in the development of allergic disorders (10–12).

## IL-1 Family of Cytokines

Although the original IL-1 family comprised only IL-1α and IL-1β, the IL-1 family has expanded considerably in the last few years and nowadays comprises 11 members (IL-1α, IL-1β, IL-1RA, IL-18, IL-33, IL-36α, IL-36β, IL-36γ, IL-36RA, IL-37, and IL-38) which have similar gene structure. All these cytokines use heterodimeric receptors for signaling. IL-1 (α and β) binds to IL-1RI, IL-33 to T1/ST2 and IL-36 (α, β, and γ) to IL-1Rrp2, and subsequently, they recruit the same coreceptor IL-1R accessory protein (IL-1RAcP). IL-18 signals through the IL-18Rα associated to the coreceptor IL-18Rβ. On receptor binding, all IL-1 family cytokines activate similar intracellular signals. The signal is initiated with recruitment of the adaptor protein MyD88 to the Toll-IL-1 receptor (TIR) domain. Several kinases are then phosphorylated, NF-κB translocates to the nucleus, and the expression of a large portfolio of inflammatory genes takes place (13) IL-1 receptor antagonist (IL-1RA) and IL-36RA, act as natural inhibitors for the biologic activities of IL-1 (α and β) and of IL-36 (α, β, and γ), respectively.

In addition to signaling receptors, also decoy receptors and inhibitory receptors for IL-1 cytokine family members had been described. One of these is the IL-1RII that does not signal because it lacks the TIR cytoplasmic domain. IL-1RII binds IL-1β with higher affinity than IL-1RI but does not transduce a signal, acting therefore as a decoy receptor (14). The IL-1RII–IL-1β complex is able to bind to IL-1RAcP, in this way the decoy receptor also serves to sequester the accessory receptor from participating in IL-1 signaling from the IL-1RI. Other receptors characterized by the ability to deliver inhibitory signals in response to IL-1 family members are SIGIRR and IL-1RAcPb (15, 16).

Each member of the IL-1 family, IL-1Ra is the unique exception, is first synthesized as a precursor without a clear signal peptide for processing and secretion, and none are found in the Golgi. IL-1α and IL-33 are similar in that their precursor forms can bind to their respective receptor and trigger signal transduction. The precursor forms of IL-18 and IL-1β do not bind their respective receptors, are not active, and require cleavage by either intracellular caspase-1 or extracellular neutrophilic proteases (17).

Since the discovery of this family of cytokines their “immunostimulant activity” was evident, but wasn’t that clear on which and how this cytokine could interact on different T lymphocytes. The present review will focalize exclusively on two members of the IL-1 family of cytokines, IL-1α and IL-1β, and it will discuss, on the basis of the last 30 years literature, their involvement in the differentiation, activation, and maintenance or survival of the different Th cell subsets.

The IL-1α precursor is produced constitutively in all epithelial cells and fibroblasts and can be also found on the surface of several cells, particularly on monocytes and B lymphocytes. The primary sources of IL-1β are the blood monocytes, tissue macrophages, and dendritic cells; B lymphocytes and NK cells also produce IL-1β (18).

## IL-1 and the Th2 Immune Response

In the 80s the first studies taking in account the direct effects of the prototypic proinflammatory cytokine, IL-1, on T lymphocytes were published (19–21). These studies indicated IL-1 as a cytokines possibly influencing growth and differentiation of immunocompetent lymphocytes. IL-1 costimulatory role for T cells was at that time attributed to two complimentary effects: (1) IL-1 can enhance transcription and secretion of the T cell growth factor IL-2; (2) IL-1 stimulates the expression of the membrane receptors for IL-2. The combination of these complimentary effects of IL-1 on T cells could explain its T-cell stimulating function. In 1988, Lichtman and colleagues (20) were the first to evaluate IL-1α costimulatory function on, at that time recently discovered, murine Th1 and Th2 cells. In this study, the authors demonstrated that only Th2 cells express high affinity receptors for IL-1 and that, accordingly, only this cell subset proliferate in response to IL-1α, whereas Th1 cells do not.

Few years later, Taylor-Robinson and colleagues (22), examining the expression of selected interleukin receptors by cloned CD4+ T cells specific for the murine malaria parasite *Plasmodium chabaudi* representative of the Th1 and Th2 subsets, found that while IL-1RI was constantly expressed by Th2 clones, its expression by the Th1 clones was either negligible or undetectable. Since then, the scientific community assumed that just the Th2 cell subset expresses IL-1RI, but lacking to confirm this data on human cells. Considering the pathogenic role of Th2 cells in allergic diseases, IL-1 activity was therefore investigated in several murine models of allergy. Nakae and colleagues (23) demonstrated that the ovalbumin-induced airway hypersensitivity response (AHR) in IL-1α/β-double deficient mice was significantly reduced when compared to wild type mice, whereas the response seen in IL-1RA-deficient mice was profoundly exacerbated, suggesting that IL-1 is required for Th2 cell activation during AHR. Accordingly, the authors showed that ovalbumin-specific IL-4 and IL-5 production by T cells, and IgG1 and IgE production by B cells in IL-1α/β-double deficient mice were markedly reduced compared with these responses in wild type mice. Similar results were obtained by Schmitz and colleagues (24) that investigated the role of IL-1 in models of allergic asthma using IL-1R1-deficient mice. The authors showed that in a model of mild asthma, based on repeated sensitization of mice with low doses of ovalbumin in the absence of any adjuvant, the pulmonary eosinophilic inflammation, the goblet cell hyperplasia, as well as antibody responses including IgG, IgE, and IgA were strongly reduced in IL-1R1-deficient as compared to wild type mice. In contrast, sensitization of mice in the presence of alum adjuvant, a more severe asthma model, rendered the IL-1 pathway dispensable for the development of pulmonary allergic Th2 responses. The role of IL-1 in sustaining the Th2 immune responses comes also from animal models of parasites infestation. Helmby and Grencis (25) showed that Th2 response-associated resistance to gastrointestinal nematode Trichuris muris is mediated was dependent on the presence of IL-1α and IL-1β. Indeed, they demonstrated that both IL-1α- and IL-1β-deficient mice were susceptible to chronic Trichuris muris infection and that the inability to eliminate the worms was associated with a defect in the development of a Th2 response in the mesenteric lymph nodes. Opposite data were obtained by Satoskar and colleagues (26) that found significantly increased IL-4 and IL-10 production by lymph node cells from *Leishmania* major infected IL-1RI-deficient mice when compared to wild type mice.

These findings are contradictory to the one showed by Helmby and Grencis, possibly because of differences in the type of cytokine/receptor KO utilized, the choice of experimental model, as well as the genetic background of the host.

The first description of IL-1RI expression and modulation on human T cells, however without distinguishing on which particular subsets, was made by Shirakawa and colleagues (21). Few years later Manetti and colleagues (27) analyzed the effects exerted by IL-1α on the growth and differentiation of human Th1 and Th2 cells. In this study, the authors showed that neither IL-1α nor the IL-1RA had detectable activity toward the antigen- or anti-CD3 antibody-induced proliferative response of already established Th1 or Th2 clones. However, allergen-specific T-cell lines, derived in the presence of anti-IL-1α Ab or IL-1RA, exhibited reduced and increased ability to produce IL-4 and IFN-γ, respectively. These data suggested that IL-1α was not required for the growth of already established human Th1 or Th2 clones, but it played a critical role in the development of Th2 cells, whereas Th1 development was unaffected. In light of the above mentioned data, the lack of an effect, described by Manetti, in terms of proliferative response to IL-1α on already established human Th2 cells and the reduction in the Th2 polarization in IL-1α neutralizing conditions, could be interpreted today as an indirect effect on non-Th2 subsets that are expanded in the presence of IL-1α (27).

The first data relative to the expression of IL-1RI on a particular Th cell subsets came out only in 2010. Indeed, Cosmi and colleagues (28) demonstrated the lack of IL-1-RI mRNA on human established Th2 clones, while Wang and colleagues (29) observed a slight membrane expression of the receptor on freshly enriched human CRTH2 positive cells, being CRTH2 a surface molecule selectively expressed by human Th2 cells (30, 31).

In any case neither Cosmi nor Wang analyzed the ability of the human Th2 cells to respond to IL-1, i.e., monitoring the activation signal transduction molecules downstream the IL-1RI, therefore it is not known if the receptor has functional activity.

Since the activity of IL-1 on human Th2 cells is not unequivocally established, caution is needed in considering this cytokine as potential new therapeutic target for human bronchial asthma as some studies suggest (32).

## Both Mice and Humans Th17 Express IL-1RI and are Modulated by Its Signaling

For the *in vitro* differentiation of naïve T cell into Th17 cells in the mouse, the scientific community was fairly unanimous in defining TGF-β and IL-6, as the key cytokines. Yet in 2006 Veldhoen and colleagues (33) described a synergistic role of IL-1β and TNF-α in the Th17 differentiation initiated by TGFβ and IL-6 and in the same year Sutton and colleagues (34) described a lower induction of Th17 cells in IL-1RI-deficient mice, than in wild type mice and also a resistance to experimental autoimmune encephalomyelitis (EAE). Interestingly, in models of autoimmune diseases, such as EAE and collagen-induced arthritis (CIA), the induction of the Th17 cells require the presence of a mixture of killed *Mycobacterium tuberculosis*, that has been recently discovered to induce, via dectin-1 and TLR4, the release of IL-1β (35). Therefore it’s possible to speculate that IL-1β plays a pivotal role in Th17 induction. This hypothesis is confirmed observing mice deficient in caspase1-enzyme that cleaves IL-1β precursor into a mature form-, where EAE is markedly attenuated. On the other hand when IL-1β activity is unopposed, like in IL-1RA knot-out mice (C57BL/6J), causes autoimmunity and arthritis that closely resembled RA in humans (36). These data have been confirmed, observing that mice specifically deficient in endogenous IL-1RA developed an increased Th17 response, and CIA appears to be because of unrestrained IL-1 activity (37, 38), which may in turn, contribute to a more severe form of CIA. In keeping with these observations Coccia and colleagues (39) showed that IL-1β promotes intestinal inflammation by augmenting the recruitment of granulocytes and the accumulation and activation of innate lymphoid cells (ILCs) in a model of in Helicobacter hepaticus-triggered intestinal inflammation. In particular, the observation that synergistic interactions between IL-1β and IL-23 sustain innate and adaptive inflammatory responses in the gut, promoting intestinal pathology, suggests that targeting IL-1β may represent a useful therapeutic approach in IBD. To further support the possibility of IL-1β play significant role not only in the induction of Th17 phenotype, but also in their expansion and homeostatic maintenance, Sutton and colleagues reported that IL-1β can promote Th17 expansion and cytokine production *in vitro* even in the absence of TCR stimulation. The mechanisms underlying these *in vivo* phenomena became more clear since it has been described IL-1RI expression first on IL-17+CD4+ T cells of SKG mice (that spontaneously develop arthritis) (40), and later on, by the demonstration that IL-1 signaling is required for the upregulation of IRF4 and RORC (two fundamental Th17 transcription factors) during the early Th17 lineage programing and to sustain its differentiation (41).

When the differentiation process, from naïve to effector cells, was analyzed in humans, a number of evidence showed soon that a predominant role was led by IL-1β, alone or in combination with other cytokines. Annunziato and colleagues (42) described the expression of IL-1RI on Th17 cell clones derived from peripheral blood (PB) and gut specimens of Crohn’s affected patients, and, accordingly, Acosta-Rodriguez and colleagues (43) was able to induce a Th17 phenotype by culturing naïve T cells in presence of IL-1β and IL-6. In particular, IL-1β was sufficient to induce the expression of RORC and production of both IL-17 and IFN-γ. Cosmi and colleagues reported that all humans IL-17-producing cells originate from CD161+ naïve CD4+ T cells of umbilical cord blood, as well as of the postnatal thymus, in response to the combined activity of IL-1β and IL-23. Confirmation that IL-1β is important in the differentiation of Th17 cells comes from studies conducted on the CD161 positive fraction of naïve CD3+CD4+ cell from the thymus as well from the cord blood of newborns where the combination of IL-1β and IL-23 allows the Th17 polarization (44, 45).

These data were recently confirmed by Lee and colleagues (46) who, demonstrated the upregulation of IL-1RI on naïve cord blood CD4+ T cell after exposure to common γ-chain cytokines (IL-7, IL-15) plus TGF-β and establishing that such condition promote the differentiation into Th17 cells upon TCR triggering and IL-1β stimulation, which is enhanced by IL-23 and blocked by IL-1RA. The same upregulation of IL-1RI was described by Raffin and colleagues (47) on PB naïve CD4+ T cell in the presence of the combination of IL-2, IL-1β, IL-23, and TGF-β.

In human disease, several clinical studies support a role for IL-1β secreted by colon lamina propria monocytes in disease activity during IBD. IL-1β levels in the colon during active phase of IBD correlated with disease activity and high levels of IL-1β were associated with active lesions (48), suggesting an important role of this cytokine in promoting localized inflammation.

A human example of IL-1β dysregulation is the heterozygous mutation of NLRP3 gene (encoding for the inflammasome component, cryopyrin) that leads to an abnormal secretion of IL-1β by monocytes, leading to different clinical inflammatory manifestations, but all hampered by inhibition of IL-1β. Indeed Lasiglie (49) analyzing 11 patients carrying this mutation (Cryopyrin-associated periodic syndromes, CAPS) observed a skewed Th17 phenotype in PB lymphocytes, as well as an increased production of IL-1β and IL-23 by monocyte-derived dendritic cells. The anti-IL-1β treatment *in vivo* reduce the secretion of IL-1β by monocytes and both IL-1β and IL-23 by monocyte-derived dendritic cells *in vitro*. The observation that IL-1RA treatment leads to a down modulation of IL-23 in PBMC of celiac patients may support the hypothesis that the over expression of IL-23 in CAPS patients is actually related to an IL-1β dependent mechanism, likely associated to the activation of the inflammasome (50). The second arm of “IL-1 system” has been enlighted in 2009 with the identification (51) of the cause of a human autoinflammatory syndrome of skin and bone in a homozygous truncating mutations in the *IL-1RN* gene that leads to the lack of secretion of this receptor antagonist (IL-1RA), and as a consequence in an unopposed IL-1 signaling. Increased number of IL-17 secreting cells was found in biopsy samples of inflamed skin from patients with deficiency of the IL-1RA (DIRA patients); as expected treatment with Anakinra, a recombinant IL-1RA, leaded to symptoms remission.

We can conclude that many evidence in mice and humans support the concept that IL-1β, acting concurrently with other cytokines, is a key cytokine in the early phases of Th17 development, acting through its specific receptor expressed already by the naïve CD4+ Th17’s precursor.

Moreover, even if IL-1β plays an important role in combating the invading pathogen as part of the innate immune response, its dysregulation is responsible for a number of autoinflammatory disorders in which Th17 cells are involved. As a consequence, its inhibition has proved therapeutically beneficial in the treatment of a spectrum of serious, yet relatively rare, heritable pathologies. This raises the possibility that anti-IL-1 therapeutics may have broader applications than previously believed, and may be utilized across diverse disease states that are linked insidiously through heightened inflammasome activity.

## Th1 “Non-Classic” Human T Cell Express IL-1RI: A New Point of View

Because of the scientific community had assumed the absence of IL-1RI expression by Th1 cells, very few works subsequently investigated the possible expression and function of this receptor on Th cell subset. Ben-Sasson and colleagues (52) were the first that described an activation effect of IL-1β on Th1 cells in a mouse model. The first data on human Th1 cells came from the study of Cosmi and colleagues mentioned before (44). In this study, the authors demonstrated that the combination of IL-1β and IL-23 was able to induce the development of Th17 cells in CD4+CD161+ cells, and also the Th1 phenotype, in both CD4+CD161+ and CD4+CD161− cell fractions. This observation leads the authors to hypothesize that also Th1 cells able to respond to IL-1β could exist. In keeping with this hypothesis, it has been recently found (53) that the CD4+CD161+ clones and inflamed tissue derived cells able to produce IFN-γ expressed IL-1RI mRNA. Interestingly, in the synovial fluid of JIA patients, the CD4+CD161+ IFN-γ-producing cells showed higher IL-1RI mRNA expression when compared to the CD4+CD161− counterpart (54). Of note, in this paper Cosmi and colleagues has highlighted the plasticity of Th17 cells showing that Th1 clones, expressing CD161 (named as “non-classic Th1 cells”), derive from an *in vivo* shifting of Th17 cells toward a Th1 phenotype. Interestingly, the authors found significantly increased levels of IL-12 in the SF of JIA patients and that Th17 cells from the PB of healthy children could be induced to shift to Th1 cells when cultured *in vitro* in the presence of JIA SF. More importantly, this effect was completely reversed by a neutralizing anti-IL-12 mAb, strongly suggesting that the shifting of Th17 cells toward the Th1 phenotype was related mainly to the activity of IL-12 present in the SF.

The late plasticity of Th17 cells to Th1 cells has been recently confirmed also in mice, where it has been found that IL-12, or the prolonged exposure to IL-23, is able to polarize Th17 cells toward the Th1 phenotype (55). Furthermore, similar results were recently reported by Nistala and colleagues (56), that showed Th17 plasticity to Th1 to be driven by the inflammatory environment in human autoimmune arthritis. Finally, very recently, the instability of the Th17 phenotype has been definitively demonstrated, at a genetic level, in mice (57).

These new data leads us to argue that up to now the scientific community overlooked this population of “non-classic Th1” cells expressing CD161 and IL-1RI (53). As mentioned before in this review many animal models of autoimmune disorders demonstrate the pivotal role of IL-1 in the pathogenesis of the disease and its relationship to Th17, but failed to look at the Th1 cells that could be affected by a lack of IL-1 signaling. In this context, the presence, and sometimes the prevalence, of Th1 cells in the inflammatory tissues have been interpreted as a protective, rather than proinflammatory, function. In humans many autoinflammatory disorders are treated blocking IL-1β [i.e., Familial Mediterranean fever (FMF), Pyogenic arthritis, pyoderma gangrenosum, acne (PAPA), CAPS, Hyper IgD syndrome (HIDS), Adult and juvenile Still disease Schnitzler syndrome, TNF receptor-associated periodic syndrome (TRAPS), Blau syndrome; Sweet syndrome, Deficiency in IL-1 receptor antagonist (DIRA)]. Initially, Anakinra (IL-1RA) was used to treat several chronic inflammatory diseases, today, these diseases are also successfully treated with neutralization by human anti-IL-1β monoclonal Abs. It’s time to speculate that the improvements observed during these treatments are not only due to the general anti-inflammatory effects and to the reduction in terms of production, survival and differentiation of Th17 cells, but also on the activity on Th1 effector cells in particular Th1 expressing IL-1RI probably derived from a Th17 phenotype (42, 53).

## Concluding Remarks

Since the discovery of the lack of IL-1RI on murine Th1 cells, most of the studies focused their attention to *in vivo* animal models of Th2 related diseases. Different models of mice either deficient for IL-1RI or for IL-1α/IL-1β were analyzed to verify the effects in the Th2 response; most of the studies agree with the idea that both cytokines promote proliferation and differentiation of Th2 cells *in vitro* and *in vivo*, but some other found no effects or even the opposite. The contradictory findings could be related to the different animal models used, the different protocol of disease induction, the different genetic background; furthermore the analysis conducted on *ex vivo* bulk cultures may induce to overestimate some observations that are actually side or indirect effects. As most of the conclusions made on the basis of animal models could have an impact in clinical practice, we would have expected to find many papers confirming or disproving these data on human cells. Surprisingly very few studies focused on the effects of IL-1α either IL-1β in human Th2 cells, and the findings do not enlighten if human Th2 cells express a functional IL-1RI and therefore can be modulated by these cytokines.

**Figure 1:**
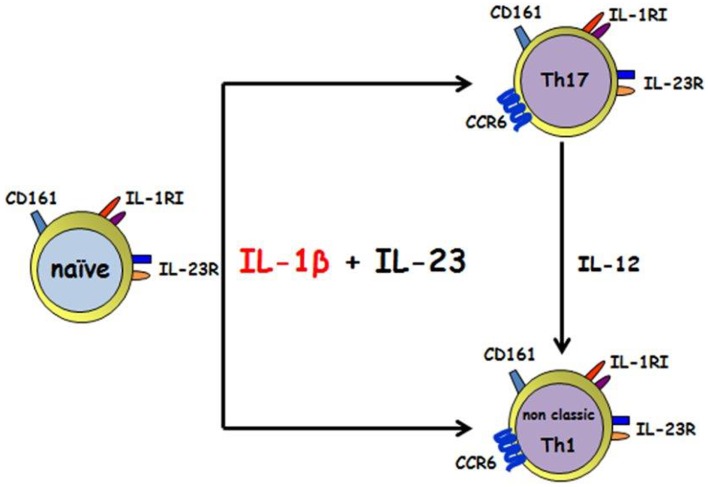
**IL-1β and IL-23 induce the differentiation of human CD161+ precursor toward both the Th17 and the non-classic Th1 phenotype**. IL-1 β together with IL-23 acts on human CD4+CD161+ precursor to induce Th17 and non-classic Th1 effector cells. Th17 can shift toward a non-classic Th1 phenotype in the presence of IL-12.

Conversely, as we look to the relationship between IL-1β and Th17, human’s studies and animal models supported both the concept that IL-1β has a fundamental role in Th17 modulation. Two genetic human diseases carrying an impairment in IL-1β either in the expression or in its regulation and showing a skewed Th17 phenotype, is for sure of great confirmation of *in vitro*/*ex vivo* data. The *in vitro* assays clarify that IL-1β is able to induce those transcription factors necessary for Th17 development, as soon as its own receptor is upregulated in naïve T cells upon TCR triggering in the presence of γ-chain cytokines. The cooperation with other cytokines, i.e., IL-23, IL-6, IL-21 leads to the differentiation and the stabilization of the phenotype (39, 43, 58, 59); IL-1RI expression is long lasting, maintained on effector Th17 cells and its signaling is probably responsible for their survival during inflammation. The recent discovery of Th17 plasticity toward a Th1 phenotype in the presence of an inflammatory environment is driving the scientific community to focus attention also to those Th1 highly present in many autoimmune diseases that have been so far considered protective rather than pathogenic; it is intriguing, that also a sub population of human Th1 cells expressing CD161 and deriving from Th17 (named “Th1 non-classic”) do express IL-1RI and most likely respond to IL-1β (Figure 1). It is therefore likely that the therapeutically approaches were the IL-1β activity is blocked, like in JIA patients, are effective because acting on these Th1 CD161+ IL-1RI cells whose number correlate with some parameter disease. Moreover other autoimmune diseases, where Th1 CD161+ IL-1RI cells were increased, could benefit of an anti-IL-1 treatment.

## Conflict of Interest Statement

The authors declare that the research was conducted in the absence of any commercial or financial relationships that could be construed as a potential conflict of interest.
